# Embedding and cross-sectioning as a sample preparation procedure for accurate and representative size and shape measurement of nanopowders

**DOI:** 10.1038/s41598-023-51094-0

**Published:** 2024-01-04

**Authors:** Paul Mrkwitschka, Bastian Rühle, Petra Kuchenbecker, Oliver Löhmann, Franziska Lindemann, Vasile-Dan Hodoroaba

**Affiliations:** 1 Division 6.1 Surface Analysis and Interfacial Chemistry, Federal Institute for Materials Research and Testing (BAM), 12203 Berlin, Germany; 2 Department 1 Analytical Chemistry, Reference Materials, Federal Institute for Materials Research and Testing (BAM), 12203 Berlin, Germany; 3 Division 5.4 Advanced Multi-materials Processing, Federal Institute for Materials Research and Testing (BAM), 12203 Berlin, Germany; 4 Division 4.2 Material-Microbiome Interactions, Federal Institute for Materials Research and Testing (BAM), 12203 Berlin, Germany; 5 Present Address: Helmholtz-Zentrum Berlin für Materialien und Energie, Hahn-Meitner Platz 1, 14109 Berlin, Germany

**Keywords:** Nanoparticles, Scanning electron microscopy, Characterization and analytical techniques, Nanometrology

## Abstract

Reliable measurement of the size of polydisperse, complex-shaped commercial nanopowders is a difficult but necessary task, e.g., for regulatory requirements and toxicity risk assessment. Suitable methods exist for the accurate characterization of the size of non-aggregated, stabilized, spherical and monodisperse nanoparticles. In contrast, industrial nanoscale powders usually require dedicated sample preparation procedures developed for the analysis method of choice. These nano-powders tend to agglomerate and/or aggregate, a behavior which in combination with an innate broad particle size distribution and irregular shape often significantly alters the achievable accuracy of the measured size parameters. The present study systematically tests two commercially available nanoscale powders using different sample preparation methods for correlative analysis by scanning electron microscopy, dynamic light scattering, Brunauer–Emmet–Teller method and differential mobility analysis. One focus was set on the sample preparation by embedding nanoparticles in carbon-based hot-mounting resin. Literature on this topic is scarce and the accuracy of the data extracted from cross sections of these particles is unclearly stated. In this paper systematic simulations on the deviation of the size parameters of well-defined series of nanoparticles with different shapes from the nominal value were carried out and the contributing factors are discussed.

## Introduction

The accurate measurement of size and shape distribution of nanoparticles from industrial powders is a complex analytical task which requires dedicated sample preparation procedures and validated measurement methods. Various sample preparation procedures exist for imaging techniques used to analyze the morphology and chemistry of raw powders dispersed in organic solvents or water, such as drop casting^1,2^, deposition by electrospraying^3^, or spin coating^4^.

While ideal, simplified model nanoparticles (monodisperse, spherical) are used for method validation with the particles serving as a reference material, further research is required with respect to the accurate characterization of industrial raw powders. These mostly show a tendency for lasting agglomeration, are complex and irregularly shaped, and are provided with a broad particle size distribution. As toxicity of nanoparticles has been reported^5,6^ for a variety of nanoscale chemical compounds, characterization steps must be developed and applied to ensure the material’s safety when dealing with derivated products. For nanomaterials, that are brought into commerce, there are several regulations that must be considered, for instance the recently introduced REACH Regulation, that relies on the EU Recommendation for a definition of a nanomaterial depending on the number-based size distribution^7^. A modified sample preparation approach based on the procedure reported by Theissmann et al.^8^ is utilized by us and systematically applied to two selected commercial nanoscale powders, namely Cerium(IV)-oxide and Zinc-oxide. The size of particles including those within agglomerates is measured by means of (i) embedding the particles within a proper matrix and conventional metallographic preparation of cross-sections of the embedded particles, followed by (ii) Scanning Electron Microscopy (SEM) imaging and (iii) analysis of the recorded images. In this way, not only a very large number (i.e. statistically relevant, at least 800 particles) of particles is contained in the cross-section SEM images, but also the representativity of all particle size fractions present in the raw powder with a potentially broad size distribution is guaranteed. Additional to SEM, the two industrial powders were analyzed by dynamic light scattering (DLS), Differential Mobility Analysis (DMAS) and Brunauer–Emmet–Teller (BET) isotherm analysis. From the SEM micrographs the particle size distributions were extracted and evaluated with respect to three different preparation methods (embedded, dispersed on a substrate and raw powder). The obtained size values were then compared to DLS results as well as to those derived from the volume-specific-surface area (VSSA) values obtained from BET measurements. The VSSA value is calculated as the product of the measured SSA values as measured with the BET analysis and the skeleton density (theoretical or measured). It should be noted that cross-sectioning through the particle agglomerates at random planes is expected to systematically slightly bias the results towards smaller particle sizes in comparison to 2D projections of the entire particles as imaged by electron microscopy with the particles deposited on a substrate. To estimate the systematic error of embedding and cross-sectioning, random cross-sections of randomly generated and oriented particles with complex shapes were simulated using a custom, self-implemented Python script.

## Materials and methods

### Chemicals

Cerium(IV)-oxide CeO_2_ and Zinc-oxide ZnO nanoparticles are available as commercial grade nanopowders. CeO_2_ was purchased from Sigma Aldrich (order number 700290) and ZnO from ChemPUR (order number 903614), with limited data supplied in the Results section under “nominal value”. We determined by X-ray diffraction (XRD) (Seifert XRD 3000 TT) that the crystal structure best matches with zincite (see Figure S1 in the Supporting Information), for both raw dry powder and the embedded powder. It will be referred to as “ZnO” throughout the paper. The database ICDD PDF-2 1993 was used for reference powder diffraction patterns. For hot mounting a carbon-based resin was purchased from Struers (Polyfast order nr. 4010036).

### Methods

#### SEM

To compare different methods with SEM (Supra40, Zeiss). The two nanopowders were prepared as-is (method ❶), in a dispersion deposited on a substrate (method ❷) and embedded in resin (method ❸):

❶ Initially, a representative amount of the raw powder was applied onto a self-adhesive carbon tab with a spatula and measured as is by SEM.
❷ For scanning transmission electron microscopy in SEM (STEM-in-SEM) the samples were prepared by wet dispersion on a conventional copper TEM grid with a carbon supporting foil. CeO_2_ powder was dispersed in a 3 mmol L^−1^ aqueous diphosphate (Na_4_P_2_O_7_) solution. ZnO was dispersed in Isopropanol (IPA/2-propanol) due to its tendency to dissolve in water. Both dispersions (solid concentration of 10 mg mL^−1^) were transferred into a reaction vial. By using a cup sonicator (BR 30, Bandelin) with active cooling (circulating thermostat LOOP L100, Lauda) the samples each were continuously sonicated indirectly for 10 min while keeping the temperature below 32 °C, followed by further vortex shaking for 10 s at approx. 1250 rounds per minute (rpm) (Vortex Mixer D-6012, neoLab Migge) and repeated indirect sonication and vortexing for a total of 60 mins. For the SEM measurement a droplet of 6 µL dispersion was applied using 0.5–20 µL tips with a piston pipette (Eppendorf) on a TEM grid (200 µm mesh carbon supported copper). Prior to drop casting the TEM grid is placed on 1 cm x 1 cm paraffin band on top of a cover glass mounted on a spin coater. The spin coater is then operated at 10000 rpm for 2 min with a ramp up time of a few seconds.
❸ To obtain a representative microsection sample for the electron micrographs of the Ceria and Zinc oxide nanopowders, the powders were embedded within a carbon-based hot mounting embedding agent. For this, they were mixed in a ratio of 1:12 (1 g nano powder to 12 g embedding agent). For homogenization, both samples were mixed for 30 mins with a tumble batch mixer (TURBULA T2 C, WAB AG). The mixture was then cast with a hot mounting device (ProntoPress-20, Struers) at 175 °C with 10 bar for 15 mins. The two microsection samples have a diameter of 30 mm and were then ground with a Piano-220 diamond disc on a semi-automatic grinding and polishing device (Tegramin-30, Struers) for approximately 2 mins to generate a plane surface. The samples were polished with a 9 µm diamond-based suspension for 9 mins on allegro/largo cloth discs. Further polishing for 5 mins with 3 µm diamond suspension on DAC discs and then 2 mins with a 1 µm diamond suspension on a Nap disc was applied. The last polishing step was carried out with a ¼ µm diamond suspension on a Nap disc. In between all the polishing steps the samples were cleaned with water and soap as well as briefly ultrasonicated.

For the micro-sectional samples of ZnO and CeO_2_ at least eight images were taken with the (secondary electron) SE InLens detector at 5 kV acceleration voltage. The chosen image format for the images used for particle size analysis is 2048 × 1536 px with a pixel size of 0.625 nm/px which corresponds to a field of view of 1280 nm × 960 nm. Further inspection on the dispersed samples was carried out in the STEM-in-SEM mode. To obtain accurate size and shape descriptors, the data was pre-processed using median filtering, then the threshold algorithm ISOData^9^ from the software package ImageJ^10^ was applied, and the binarized images were post-processed manually. From the regions of interest the size descriptors minimum Feret diameter and the area equivalent circular diameter (ECD) were extracted^11^.

#### DLS

Samples for DLS were prepared as wet dispersion as described in the SEM section according to the method denoted ❷. After 60 min vortexing at 1250 rpm and sonication, the CeO_2_ sample was centrifuged for 36 s (short spin mode) at 100 g with a centrifuge (MiniSpin® plus, Eppendorf). Afterwards, 500 µL of the supernatant were carefully pipetted with a piston pipette (Eppendorf) into a new vial and measured. The ZnO-Isopropanol dispersion was left to sediment for 60 min before measurement. Here again, the supernatant was carefully taken from the sample and measured.

To determine the particle size of the dispersed powder samples (see sample preparation method ❷), DLS (NanoFlex, Microtrac) was applied according to ISO 22412:2017^12^. The instrument is operated in heterodyne mode with a wavelength of 780 nm under a measurement angle of 180°. The evaluation of the results was performed using frequency analysis utilizing the following refractive indexes: n_water_ = 1.33, n_isopropanol_ = 1.39, n_CeO2_ = 2.42 and n_ZnO_ = 1.99. The refractive indices were used to obtain a volume-weighted particle size distribution by means of the Mie-correction.

#### BET (VSSA)

For gas sorption and density measurements the powder samples were dried at 300 °C for 3 h under vacuum (< 0.1 Pa) before measurements. To test the plausibility of the SEM results, a comparative analysis by the VSSA-method, described in detail elsewhere,^13^ was performed. VSSA is derived from the product of the specific surface area (SSA) obtained with the BET method and the skeleton density ρ according to Eq. (1):

1 $$VSSA\left[ {\frac{{{\text{m}}^{{2}} }}{{{\text{cm}}^{{3}} }}} \right] = SSA\left[ {\frac{{{\text{m}}^{{2}} }}{{\text{g}}}} \right]\rho \left[ {\frac{{\text{g}}}{{{\text{cm}}^{{3}} }}} \right]$$

Assuming monodisperse, spherical, nonporous particles, the diameter of the particles can be derived from this VSSA value using Eq. (2):

2 $$d_{VSSA} \left[ {{\text{nm}}} \right] = \frac{6000}{{VSSA\left[ {\frac{{{\text{m}}^{{2}} }}{{{\text{cm}}^{{3}} }}} \right]}}$$

In the case of the present powder samples, the electron micrographs shown in Fig. 2 show irregular shaped particles, with a broad size distribution, which implies that the diameter derived from the VSSA can only be assumed as an approximate value.

The SSA was obtained by means of Ar-gas sorption at 87 K instead of using nitrogen gas (Autosorb iQ, Quantachrome) as recommended for metal oxide powders such as ZnO and CeO_2_^14^. The skeleton density was determined by He-pycnometry (AccuPyc II 1340, Micromeritics) according to ISO 12154:2014^15^.

#### DMAS

75.5 mg of Cerium oxide were weighted on a glass plate. 5 drops of ethanol were added, and the dispersion was mixed with a spatula. Afterwards, 7 drops of Na_4_P_2_O_7_ solution (3 mM) were added and mixed again with a spatula. The dispersion was qualitatively transferred into a 2 mL vial and 1.5 mL of Na_4_P_2_O_7_ solution (3 mM) were added. Final dispersion was achieved by a cup sonicator (BR 30, Bandelin) with active cooling (circulating thermostat LOOP L100, Lauda). The sample was continuously sonicated indirectly 10 times for 10 min with cooling time in-between keeping the temperature below 32 °C. The samples were measured at latest 24 h after preparation. For that, 300 μL of the supernatant were diluted in 700 μL Na_4_P_2_O_7_ solution (3 mM). The dilution was treated in an ultrasonic bath for 10 min directly before the measurement. Diluted samples were transferred to an electrospray aerosol generator (Model 3482, TSI). Here, the grounded sample was transferred to an aerosol by applying an electric field which created a Taylor cone at the end of a glass capillary (ID 25 μm), resulting in a release of individual droplets. A mixture of dried air and CO_2_ was used as a carrier gas. The solvent evaporates and all non-volatile components form airborne particles. The pressure on the sample was adjusted to a constant flow resulting in a stable Taylor cone. Air and CO_2_ flows were adjusted to 1.42 L/min and 0.08 L/min, respectively. The setup for aerosol generation is based on the setup described in^16^. The sample was moved to the capillary through microfluidic tubes and filtered by a 2-micron inlet filter to avoid capillary clogging. The airborne particles passed an X-ray neutralizer, were size selected by an electrostatic classifier (Model 3082, TSI) equipped with a differential mobility analyzer (Model 3081A Long DMA, TSI), and, finally, counted by a condensation particle counter (Model 3776, TSI). The sample flow rate and sheath flow rate were set to 1.5 L/min and 15 L/min, respectively. Each measurement took 5 min at a resolution of 64 channels per decade. The spectra were corrected for diffusion loss and multiple charge. The analysis was carried out on an average of 5 spectra. Zinc-oxide dispersed in IPA was not measured due to its low conductivity.

### Geometrical simulation of particles for 3D size measurement of embedded particles by sequential slicing

Simulations of cross-sections for size measurements with SEM of isotropic and anisotropic particles were done with a custom, self-implemented Python script (Python 3.8 with the help of the packages matplotlib, numpy, opensimplex, and trimesh). In brief, we started from an Icosphere base mesh with radius 1 and 10,242 vertices that was isotropically scaled by a random factor drawn from a gaussian distribution with mean 1 and standard deviation 0.1 to simulate particle polydispersity. For anisotropic particles, the resulting mesh was further scaled along the x and y directions by random factors drawn independently from uniform distributions between 0.6 and 0.7 to introduce the anisotropy. Next, 3D OpenSimplex noise was generated and added to each vertex coordinate to simulate bumps and dimples found on the surface of real particles (the noise generator was randomly seeded, and a noise frequency of 0.6 and noise amplitude scaling of 0.3 were used). Afterwards, anisotropic particles were rotated in spherical coordinates by random angles theta and phi (sampled from independent uniform distributions in the range from 0 to π and 0 to 2π, respectively) to remove the bias along the z-direction that was introduced earlier during the anisotropic scaling in x and y directions of the base mesh. This random rotation was omitted for isotropic particles for performance reasons (here, all scalings and noise functions that were applied before were completely isotropic, i.e., there should not be any bias or preferential direction and hence also no need to further randomize particle orientation in these cases). Finally, the particles were further scaled by a factor of 50 (purely for convenience since this would result in the original Icosphere base mesh having a radius of 50, respectively a diameter of 100), and a 3D mesh was constructed from the vertices using the original vertex connectivity of the Icosphere. For the 3D shape descriptors, the volume of the mesh as well as the minimum and maximum extent of its oriented bounding box were calculated. For the 2D slices, the intersection of the mesh with a 2D plane having the z axis as its normal vector and a z-offset randomly sampled from a uniform distribution between 0.99 times, the smallest and largest vertex coordinates of the mesh along the z-axis were calculated; further, the area as well as the extent in x and y directions of the oriented bounding box of the 2D slice were extracted. It should be noted that even though the z-axis was arbitrarily chosen as the normal direction of the slicing plane, this does not introduce any bias or preferential direction due to the isotropy and randomized orientation, respectively, of the mesh.

**Figure 1:**
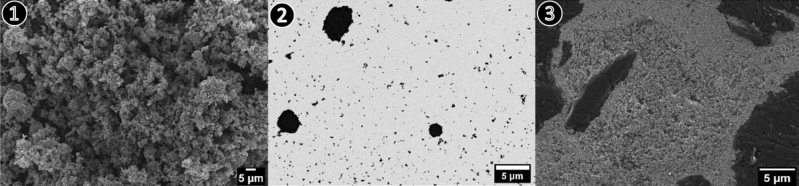
SEM micrographs of zinc oxide raw powder prepared by sample preparation method ❶ (dry powder), method ❷ (dispersion) and method ❸ (embedding) at low magnification. Micrographs ❶ and ❷ show the background in black and bright particles whereas in ❸ the image shows the particles in black with a bright background.

**Figure 2:**
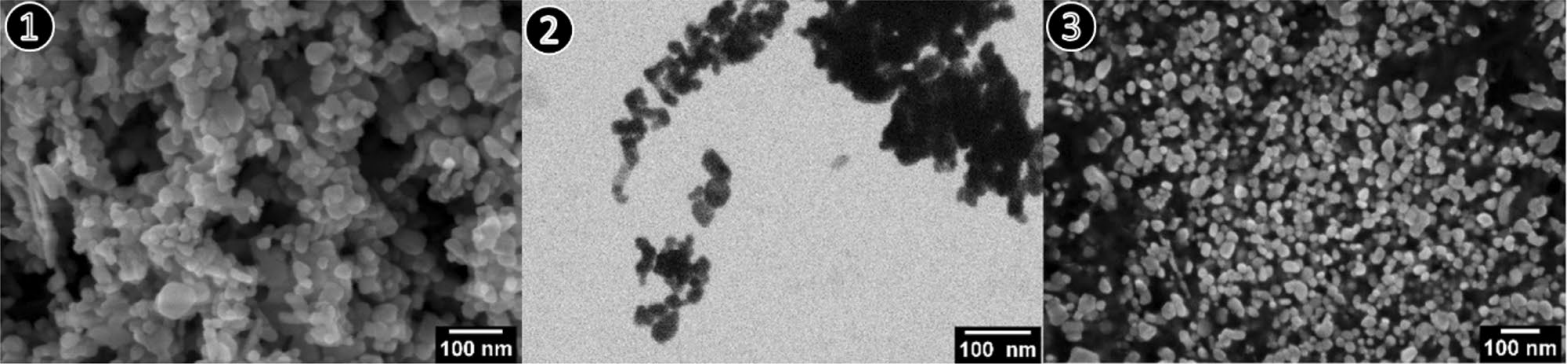
SEM micrographs of zinc oxide raw powder prepared by sample preparation method ❶ (dry powder), method ❷ (dispersion) and method ❸ (embedding) at high magnifications. Micrographs ❶ and ❷ show the background in black and bright particles whereas in ❸ the image shows the particles in black with a bright background.

**Figure 3:**
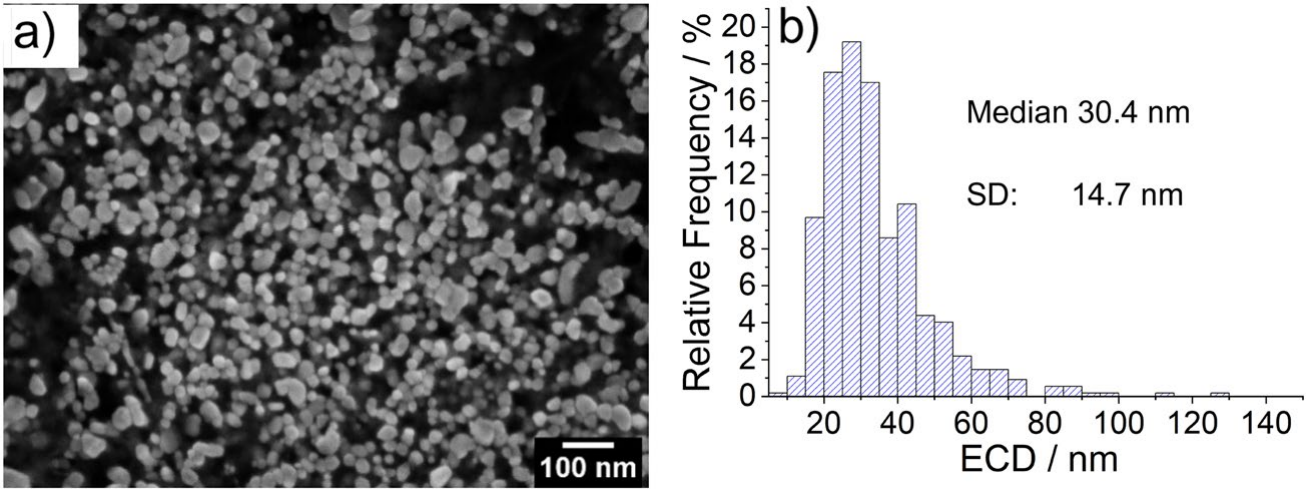
SEM micrograph of embedded zinc oxide nanoparticles after cross-sectional preparation (**a**) with the extracted particle size distribution (PSD) of the size descriptor equivalent circular diameter (ECD) (**b**).

**Figure 4:**
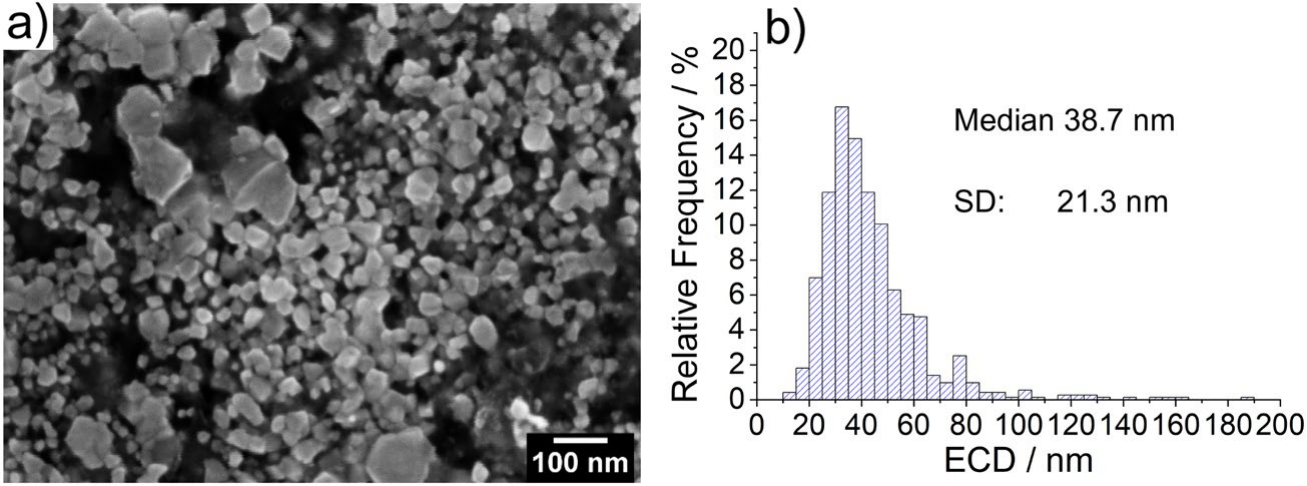
SEM micrograph of embedded ceria nanoparticles after cross-sectional preparation (**a**) with the extracted particle size distribution (PSD) of the size descriptor equivalent circular diameter (ECD) (**b**).

**Figure 5:**
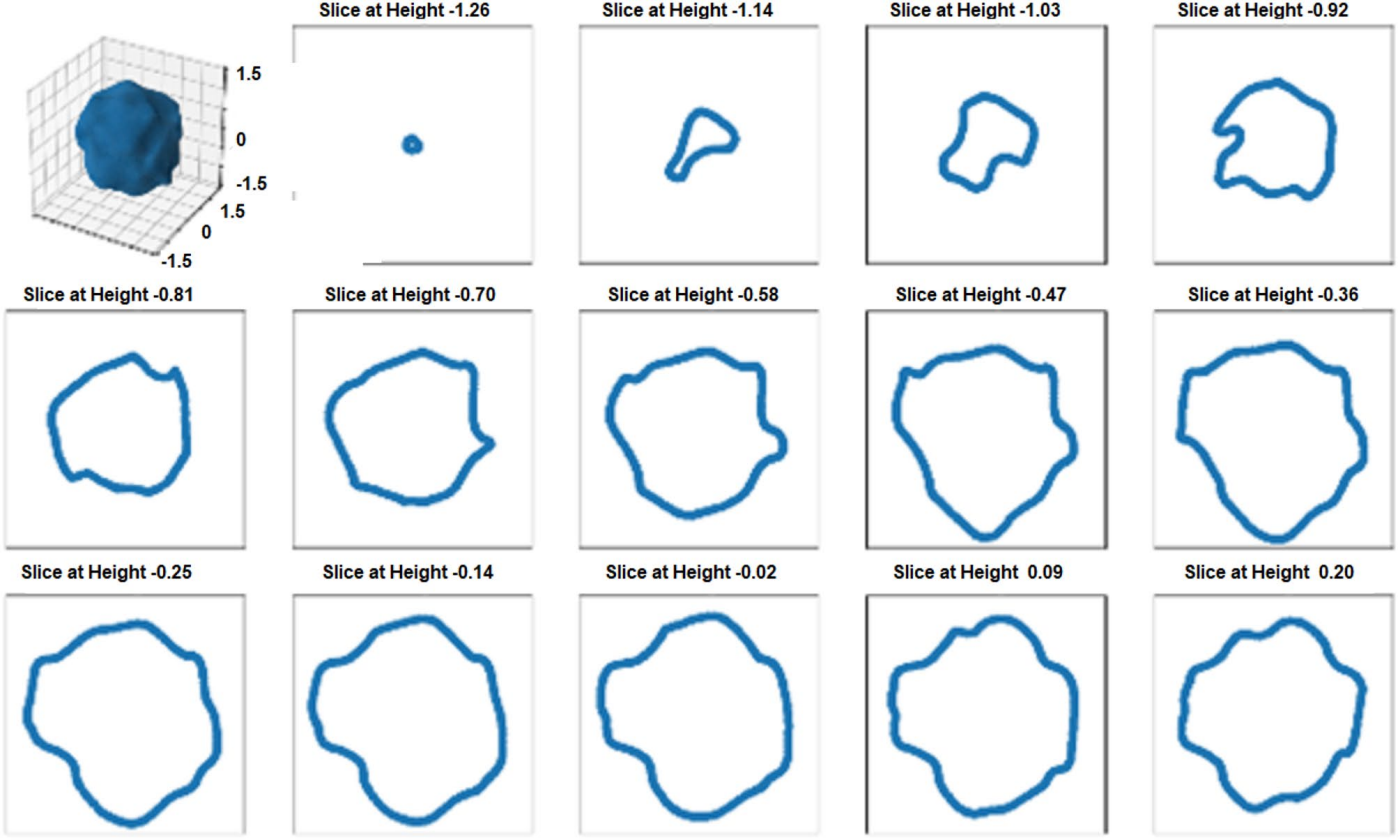
Simulated isotropic particle and the corresponding sequential cross-sections of the lower half of the particle along one direction.

**Figure 6:**
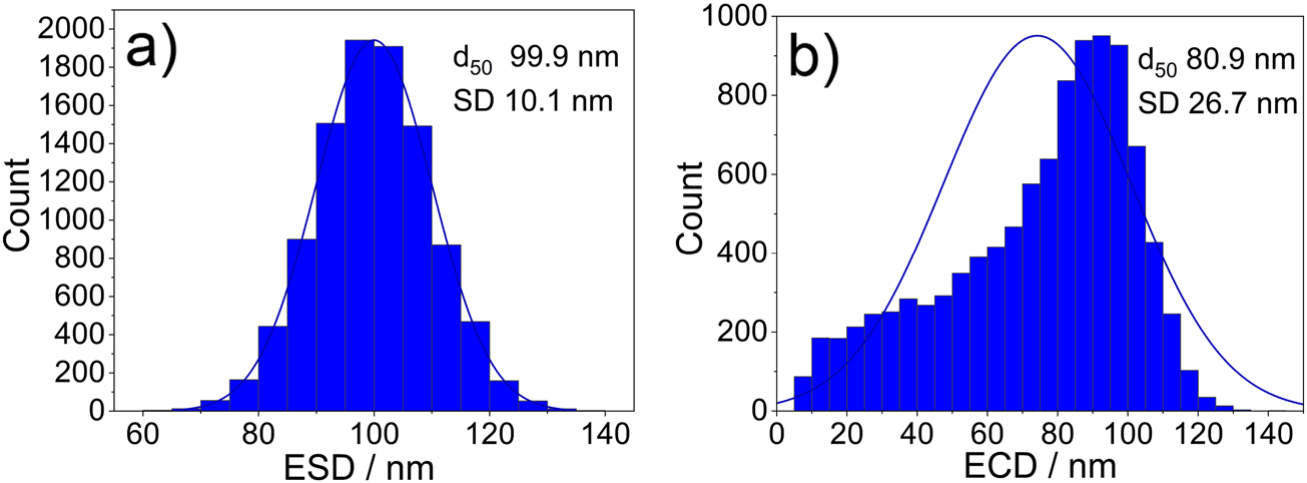
Input ESD (**a**) and the calculated ECD (**b**) of random cross-sections of simulated 10,000 isotropic particles to assess the theoretical accuracy of the embedded nanoparticle preparation method.

It should be noted that the choice of all hyperparameters for randomly generating the particles, such as a standard deviation of 0.1 for the “size polydispersity”, an “anisotropy factor” between 0.6 and 0.7, and a “noise frequency” of 0.6 and “noise amplitude scaling” of 0.3, were completely arbitrary and are not based on any real measurements or properties of the samples shown in Figs. 1, 2, 3 and 4. Hence, a comparison of the simulation and the real results can only be qualitative. Exemplary size descriptors as well as 3D particle shapes and 2D sections through these particles are shown in Figs. 5 and 6.

## Results

### SEM

The three different sample preparation methods for SEM analysis as described above are presented in detail and their performance regarding accurate measurement of the nanoparticle size distribution is comparatively evaluated: (i) raw powder was measured as received upon shipping (method ❶), (ii) method ❷ with the particles over liquid dispersion deposited on a substrate (TEM grid), and (iii) nanoparticles-embedded-in-resin (method ❸).

In Fig. 2 the results of different sample preparation methods are visualized at two different magnifications. Method ❶ in Fig. 2 shows the powder in its initial state in detail. The overview image for Method ❶ (Fig. 1) depicts predominately agglomerated/aggregated particles. For the dispersed nanoparticles (method ❷, Fig. 2) the raw powder has undergone significant pre-processing steps including dispersing, sonication, deposition on a substrate and drying. The overview image of method ❷ (Fig. 2) shows that large agglomerations in the micrometer scale are still present, and the dispersing process was not completely successful. The SEM micrographs of the embedded nanoparticles for both magnifications (method ❸, Fig. 2) illustrate homogenously distributed particles after cross-section preparation.

The result for the embedded samples consisting of 8 micrographs for each type of material with at least 800 particles counted are shown by a representative image in Figs. 3 and 4 with the corresponding particle size distribution (PSD) of the size descriptor equivalent circular diameter (ECD). Results for size values obtained from micrographs prepared by method ❷ are given in the SI as Figure S2. A complete summary of the results with the size descriptors MinFeret and ECD is given in Table 1.

**Table 1:** Sizes of CeO_2_ and ZnO as measured with SEM, STEM-in-SEM, BET, DLS and DMAS.

Method	Measurand	CeO_2_	ZnO
		Value/nm	
SEM (method ❸)	d_MinFeret,50,0_	34.7	27.1
	d_ECD,50,0_	38.7	30.4
STEM-in-SEM (method ❷)	d_MinFeret,50,0_	25.3	20.2
	d_ECD,50,0_	29.1	22.5
VSSA	d_VSSA_	32	42
DLS	d_50,3_ (d_50,0_)	63*(39)	200*(132)
DMAS	D_50,0,emob_	37.4	–**
Producer information	Nominal value	< 50	20

*The median ECD extracted (as an approximation) from the calculated number-weighted particle size distribution.

**Not available, as ZnO was dispersed in IPA and thus it was not measurable with DMAS due to low conductivity.

### DLS

The DLS results are displayed in the SI (see Figure S3). For CeO_2_ and ZnO a median diameter of 63 nm and 200 nm are obtained, respectively. In the case of ZnO, the small contributions in the right wing of the PSD in Fig. 3 indicates presence of agglomerates/aggregates. The displayed DLS results are volume-weighted and, therefore, not directly comparable to the number-weighted size distributions obtained with electron microscopy and DMAS.

### BET

The BET results obtained by means of Ar gas adsorption are SSA_BET_ = 27.2 m^2^/g for CeO_2_ and 26.3 m^2^/g for ZnO. The skeleton density amounts to *ρ* = 6.84 g/cm^3^ for CeO_2_ and 5.44 g/cm^3^ for ZnO. Thus, by means of Eq. (2) a “screening” diameter of 32 nm for CeO_2_ and 42 nm for ZnO are finally obtained.

### DMAS

In the SI Figure S4 the presented data are fitted with a lognormal distribution showing a broad particle size distribution with the median value at 37.4 nm and a standard deviation of 1.61.

The results for the two nano powders as measured with all the methods SEM, STEM-in-SEM, BET/VSSA, DLS and DMAS are tabulated in Table 1.

### Geometrical simulation of complex shape particles for 3D evaluation of size by sequential slicing

The sequential cross-sectioning along one direction through randomly generated isotropic particles was simulated and is shown as an example for one particle together with its 3D image in Fig. 5.

The distribution of the volume equivalent spherical diameter (ESD) of randomly simulated 10,000 isotropic particles with a median of 100 nm and a standard deviation of 10 nm is plotted in Fig. 6a. The result of the random slicing of these 10,000 particles as they would be imaged by SEM on cross-sectionally prepared samples is expressed in Fig. 6b as the PSD of the ECD.

A difference of 19% between the ESD and the ECD for the median values was observed. Note that in contrast to the normal ESD distribution, the ECD distribution is skewed, with a modal value of ~ 92.5 nm and a median of 80.9 nm and a corresponding larger SD.

**Figure 7:**
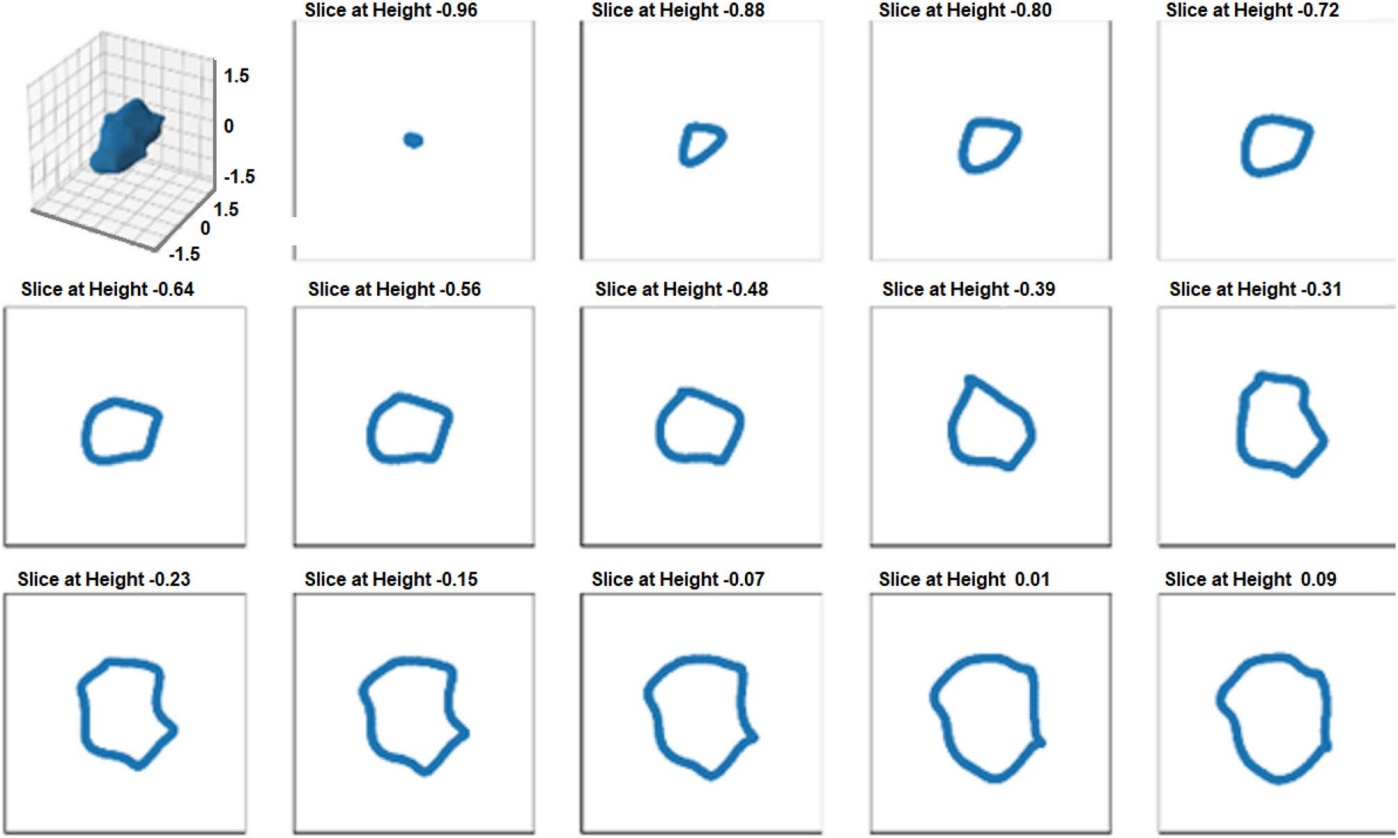
Simulated anisotropic particle and the corresponding sequential cross-sections of the lower half of the particle along one direction.

An image representatively depicting the randomly generated anisotropic particles and corresponding cross-sections are depicted in Fig. 7.

**Figure 8:**
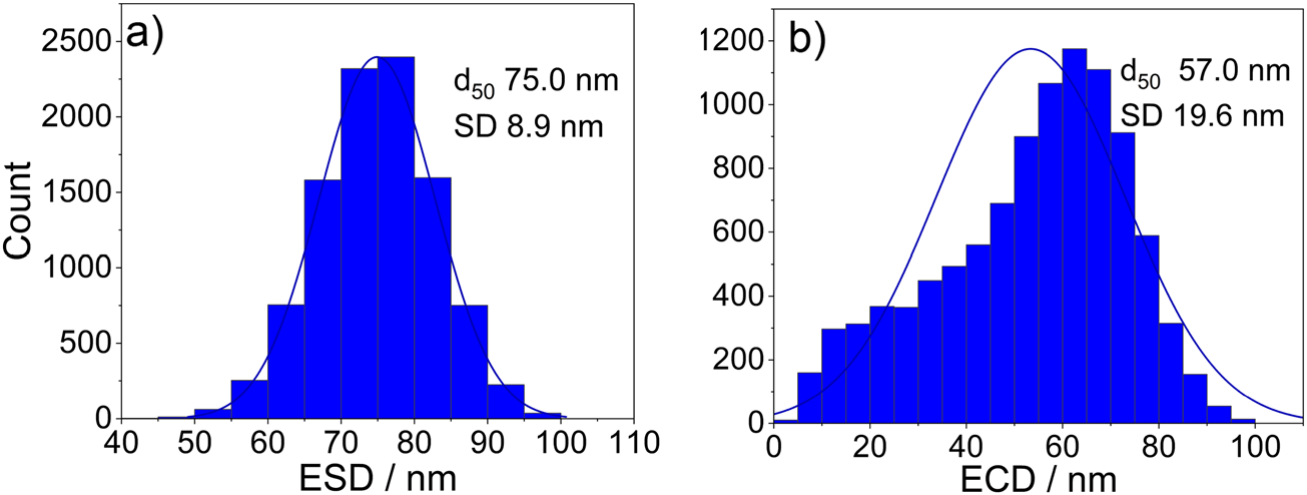
Input ESD (**a**) and calculated ECD (**b**) diameters of 10,000 random cross-sections of simulated anisotropic particles to assess the theoretical accuracy of the embedded nanoparticle preparation.

The distribution of the ESD and the ECD of cross-sections of randomly simulated particles are plotted in Fig. 8 for the case of an anisotropic particle.

When comparing the median ECD with the median ESD for the anisotropic particles, a difference of 24% is observed. Same skewness toward lower values as for the isotropic case is observed in the ECD distribution.

## Discussion

The embedding technique outlined in this study is a suitable method to measure the size and shape of commercial nano-powders in the as-received dry/non-dispersed state. Further, the agglomeration degree can be also assessed with a higher confidence by comparing the direct visual observation on a large number of particles with the size result as measured with other ensemble methods. The determined primary particle size and the size of remaining agglomerates/aggregates are particularly relevant for regulatory characterization. To characterize nanoparticles regarding their final application such as paints, sunscreen or regarding their toxicity to the human body and environment, it may not be valid for comparison as these nanoparticles are dispersed in specific delivery media other than the resin used for hot mounting. The significance of the obtained results might therefore be limited in those fields, however, the embedding procedure in this work provides a promising starting point: In this regard, the DLS method in conjunction with electron microscopy image analysis (by e.g. drop casting or spin coating) might be better suited to evaluate dispersed particles by adjusting the delivery media to the specific requirements (for instance in nanotoxicology tests).

The raw powder in its initial state (Method ❶) allows for a qualitative statement regarding the size and shape of the nanomaterial, but an accurate and representative quantitative analysis of the particle size and shape is not feasible due to the very high agglomeration/aggregation degree of the bulk powder. For the dispersed nanoparticles (Method ❷) it is possible to obtain reasonable information on size and shape, but large agglomerations on the micrometer scale are still present in the ZnO sample, so that the representative significance of the measured dimensions is affected (see Fig. 2 method ❷). Complete deagglomeration of nanopowders in dispersion is very time-consuming and sonicating the sample might even lead to further agglomeration^17^. When measuring the primary particle size by DLS (preparation described under methods section) the sample is extracted from the supernatant and thus may not be representative for the whole starting material due to sedimentation of larger particles, agglomerates, and/or aggregates. The SEM micrographs of the embedded nanoparticles (Method ❸) show particle cross-sections which could be accurately analyzed with representative statistics of at least 800 particles per nanomaterial. However, based on results obtained from the simulation of (arbitrary) isotropic and anisotropic particles, sizes obtained from cross-sectioning of embedded particles via SEM imaging showed a tendency to be underestimated (up to 24% in the simulations). This is due to the improbability to section all particles through the plane corresponding to the largest particle area. For the case of a simulated isotropic particle, a size deviation of 19% between the calculated ESD and ECD was found (see Fig. 6), which is close to the theoretically expected value of 1-π/4 ≈0.215 resulting from the analytical solution of the average radius of a cross section through a perfect unit sphere (with no added noise or polydispersity).The SEM analysis of the two different preparation methods ❷ (dispersion) and ❸ (embedding) shows a significant difference of the ECD of about 25%, with lower values corresponding to method ❷. Further, via comparison of the results of the ensemble methods VSSA (for both materials), DLS and DMAS for CeO_2_ versus the single-particle-counting method electron microscopy, the plausibility and representativeness of the results are demonstrated. The CeO_2_ results derived from DLS and DMAS are in good agreement with the SEM results (ECD) when method ❸ (embedding) is used. For comparability purposes, the volume-weighted distribution measured with DLS was converted into a number-weighted distribution, underlining that this conversion based on different assumptions is only an approximation. The DLS and DMAS results for CeO_2_ show a difference of 3% when compared to the dispersed nanoparticles measured with the STEM-in-SEM operation mode (method ❷). The diameter obtained with the VSSA-by-BET method shows the largest difference of 21% for CeO_2_. For the dispersion of the ZnO powder (i.e. method ❷) the separation of the individual particles from agglomerates was unsuccessful; this has led to the particularly large difference in particle size measured with DLS of over 300% (which is very agglomerate-sensitive) relative to the results obtained by the other techniques. The size of the constituent particles of the ZnO powder deviates significantly depending on the utilized analytical method. To summarize, all the methods SEM ❷ (dispersion), ❸(embedding), DMAS and VSSA-by-BET basically provide the size of the constituent particles, whereas the DLS-derived results are heavily influenced by agglomeration/aggregation (as shown in Fig. 2 method ❷) and, thus, show a much larger particle size as a result.

Effects which are inherent to the analysis with an electron microscope must be taken into consideration. As reported by Crouzier et al.^18^, an overestimation of the particle size when measuring it with an SE InLens detector at higher acceleration voltages in a SEM is to be considered. Further, the primary electrons penetrate the surface of the embedded samples and produce SEs in a (sub-)micrometer depth, but only secondary electrons from a depth of up to a few nanometers are emitted. The penetration depth depends on the acceleration voltage and the mean atomic number (Z) of the sample material: it increases with higher kV and with lower Z; thus, diffusing through the surrounding carbon-based embedding agent more easily than the nanoparticles. If a particle is cut above a larger sectional area, the particle size measured by means of SEs will be larger than the actual area in the plane of the cross-sectioned particle due to the penetration depth. For the opposite case when the particle is cut below a larger area the penetration depth will have no significant effect on the measured cross-sectional area. To summarize the discussed effects might lead to a slight overestimation of the results. The theoretical change in size distribution for more elongated particles (aspect ratio ~ 2:3) as compared to spherical particles can be observed when comparing the distributions in Figs. 6 and 8. The relative error of the mean equivalent diameter is similar in both cases, namely 26% (isotropic) vs. 29% (anisotropic). For the mean minimum Feret Diameter, the relative errors were 30% (isotropic) vs. 27% (anisotropic), for the mean maximum Feret Diameter, the relative errors were 29% (isotropic) vs. 39% (anisotropic). Nanoparticles can appear in many shapes, with different aspect ratios, for instance as star- or needle-shaped particles, this potentially leading to an even larger measurement uncertainty compared to the case of anisotropic particles.

## Conclusions

SEM imaging of embedded nanoparticles after careful cross-sectioning allows accurate analysis of a representative number-based size-distribution as well as shape analysis. Good agreement of the SEM results with those obtained with the ensemble methods DMAS, DLS and VSSA-by-BET (within one standard deviation) validates the proposed sample preparation method for accurate and representative nanoparticle size measurement of complex shape and broad size dispersity. The number of measured particles can be significantly increased with suitable automated analysis through AI-based segmentation algorithms, for instance as reported by Rühle et al.^19^. By applying sequential metallographic cross-section preparation and SEM analysis on the same sample it is possible to obtain depth information on a substantial number of particles. Further, by FIB (focused ion beam)-SEM tomography this preparation method offers the possibility to obtain 3D images of the analyzed region of interest. For regulatory purposes aiming at registration of nanomaterials, the reported median MinFeret together with the associated quantifiable underestimation caused by the sample preparation method via micro-sectioning provide a sufficient safety margin to reliably assess whether the studied particulate materials qualify as nanomaterials. The degree of underestimation of particle sizes measured with the proposed sample preparation method, was estimated by simulated cross-sections of arbitrary isotropic and anisotropic particles. Limitations of the method arise due to the hot mounting at 448 K which excludes the preparation of temperature-sensitive materials. Embedding organic materials such as polymers in a predominately carbon-based matrix is problematic considering the weak contrast between materials with nearly identical average atomic numbers (or density) of the materials. The performance of the embedding method for organic materials is planned to be assessed systematically further in a separate study.

### Supplementary Information

Supplementary Information.

## Data Availability

The analyzed and referenced datasets are available on request from the corresponding author.
