# Supplementary material for: Embedding and cross-sectioning as a sample preparation procedure for accurate and representative size and shape measurement of nanopowders
Source: Sci Rep. 2024 Jan 4;14:511. doi: 10.1038/s41598-023-51094-0 (PMC10766598; doi:10.1038/s41598-023-51094-0)
Supplement: Supplementary file 1 — Supplementary Information. [file 41598_2023_51094_MOESM1_ESM.docx]

**Supporting Information**

**Embedding and cross-sectioning as a sample preparation procedure for accurate and representative size and shape measurement of nanopowders**

Paul Mrkwitschka*, Bastian Rühle, Petra Kuchenbecker, Oliver Löhmann, Franziska Lindemann and Vasile-Dan Hodoroaba*


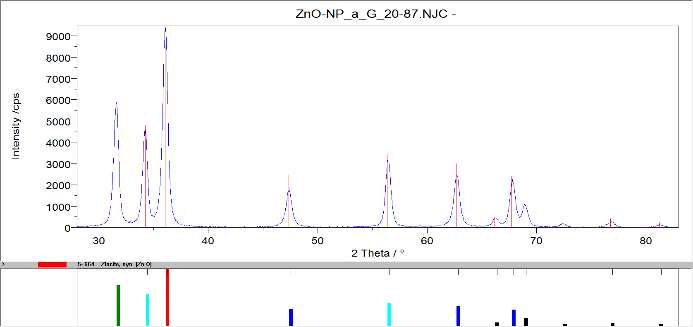

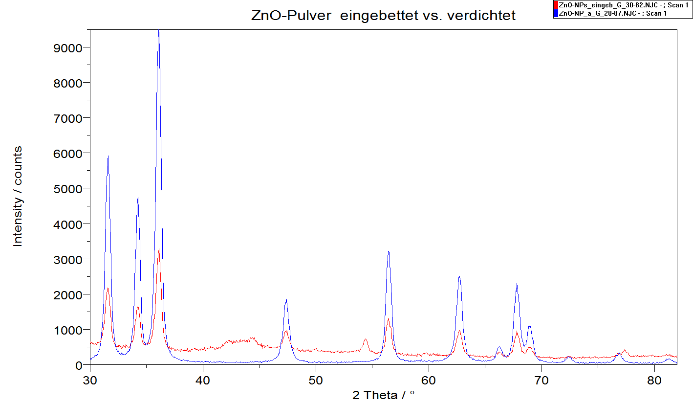


b)

a)

Figure S1: X-ray Diffraction (XRD) analysis of the dry powder as-received ZnO (from ChemPur) (a) and embedded sample (red line) compared to the dry powder as-received (blue line) (b)

The crystal structure of the ZnO dry-powder was determined by X-ray diffraction, see Figure S1, a) as best matching with zincite. Further, the embedded sample measured with XRD confirms this finding, see Figure S1 b). XRD analysis was carried out on a Seifert XRD 3000 TT diffractometer utilizing the database ICDD PDF-2 1993.

Figure S2: STEM-in-SEM micrographs showing the dispersed CeO_2_ sample (a) and the ZnO (b)


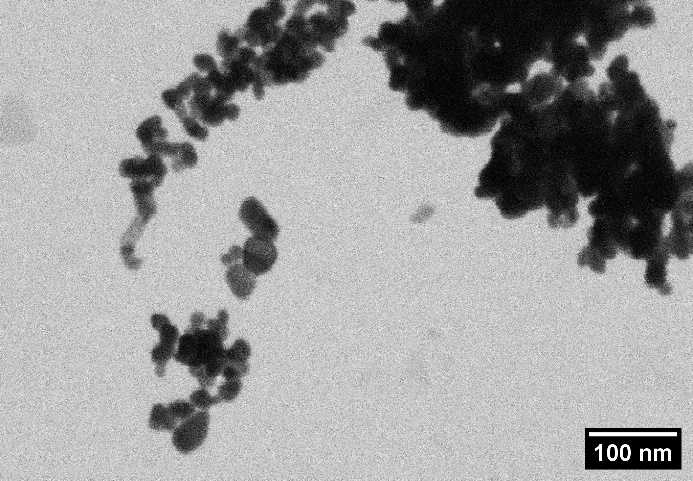

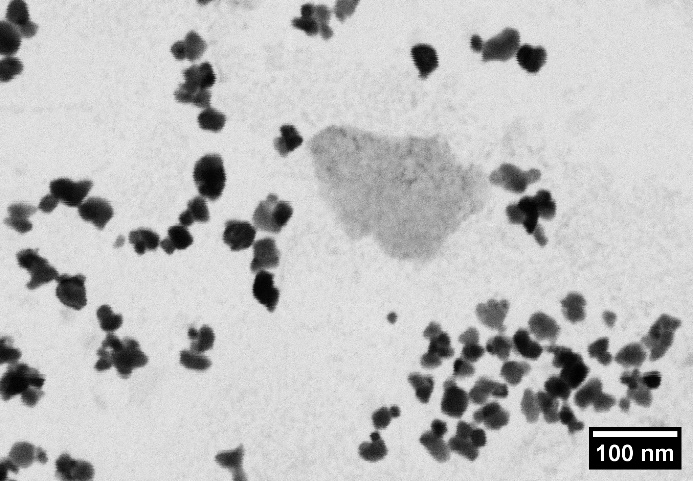


b)

a)

The comparison of ceria and zincite particles with two representative images after preparation (as described in the manuscript as method ❷) is shown in Figure S2.

For the dispersed CeO_2_ the median ECD found with SEM is 29.1 nm. The ZnO particles are predominantly agglomerated and/or possibly aggregated and, therefore, the number of single particles to be counted is limited. Thus, the resulting median ECD of 22.5 nm for ZnO must be considered as a rough estimate.

**DLS**


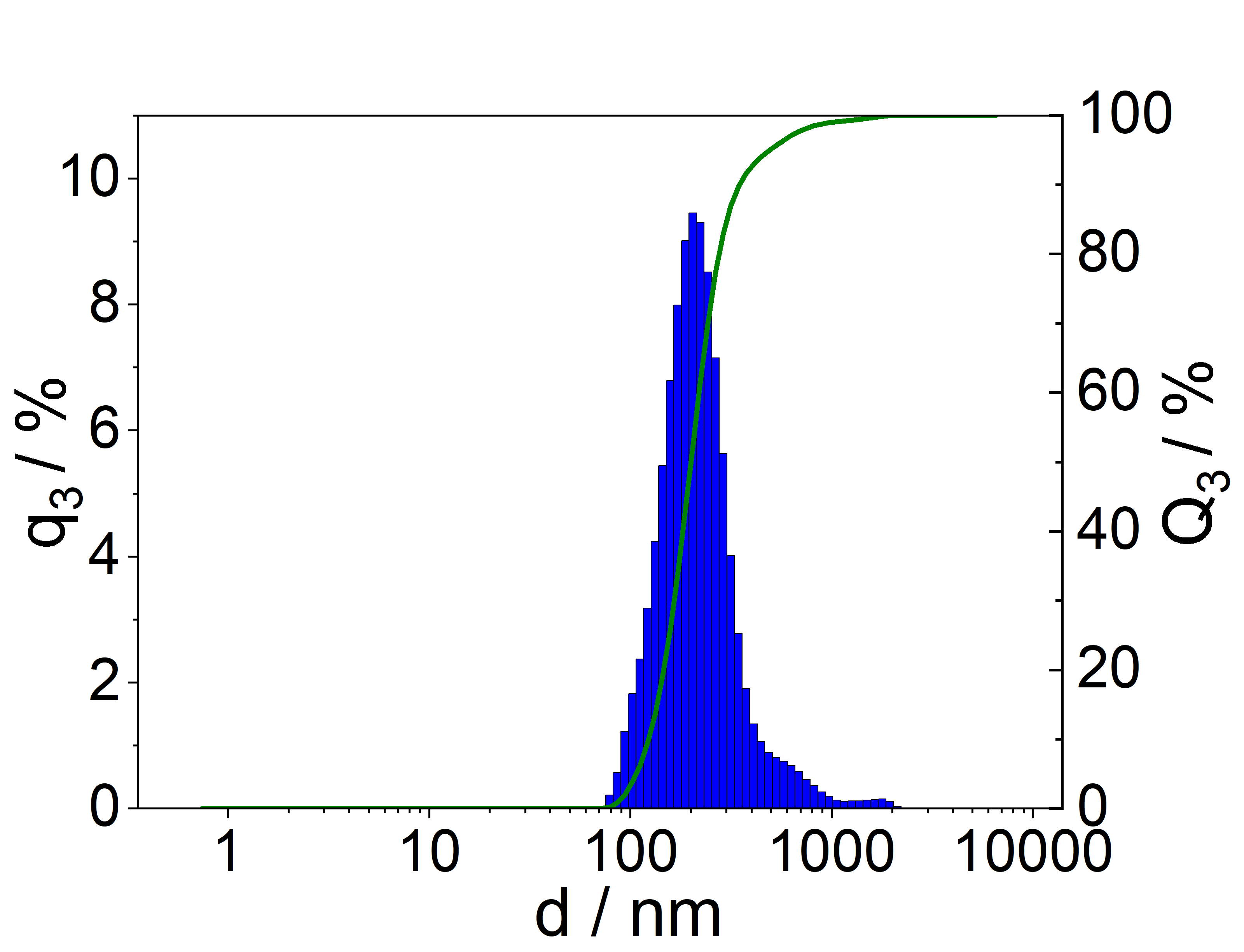

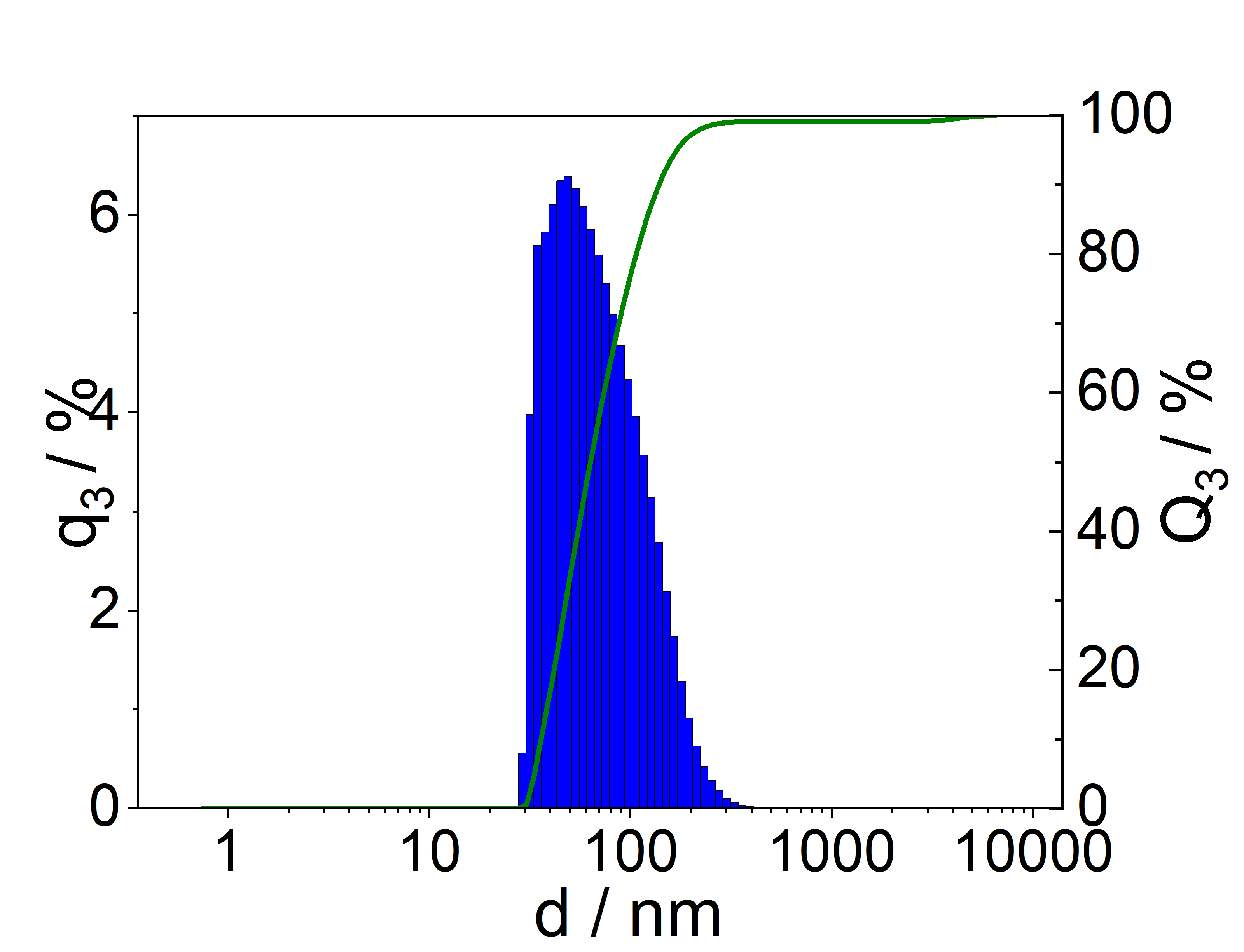


a) b)

Figure S3: DLS results of dispersed ZnO powder (a) and CeO_2_ powder (b)

**DMAS**


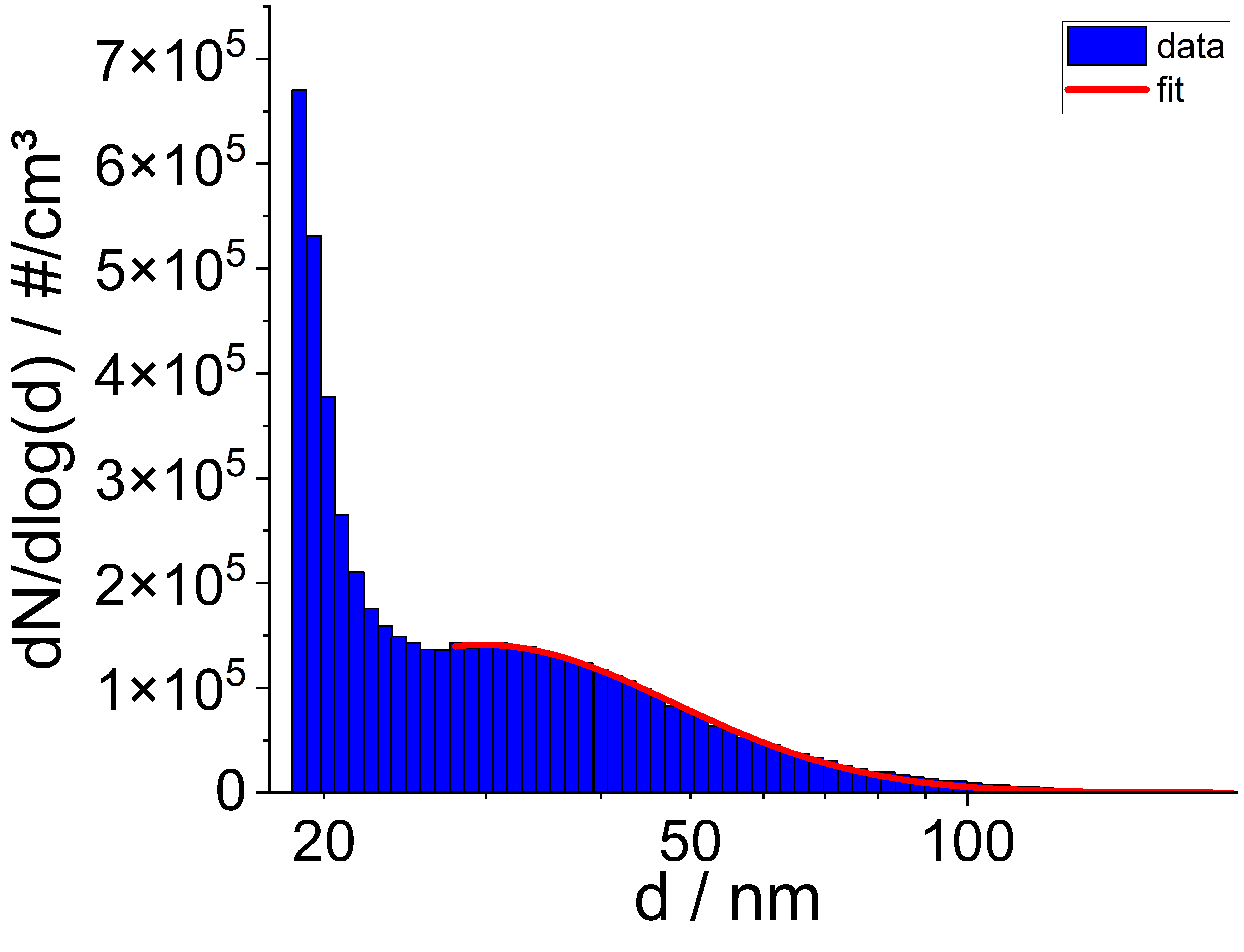


Figure S4: DMAS particle size distribution of cerium oxide as measured (blue bars) and its lognormal distribution fit (red line).

Below 18 nm a solvent peak is partially visible for the CeO_2_ sample as measured by DMAS. It results from non-volatile compounds within the dispersion other than the ceria nanoparticles e.g., Na_4_P_2_O_7_. This type of contribution depends on the initial droplet size and is also visible when measuring only the Na_4_P_2_O_7_ solution (data not shown). Thus, only data points above 18 nm are considered as valid for fitting. No particles were detected above 100 nm. So, there is no evidence of agglomerates constituted of only a few particles being present in the supernatant after sample preparation. Larger agglomerates might exist but are either sedimented in the dispersion vessel or/and are withheld by the impactor installed at the SMPS^TM^ entrance.
